# Cathelicidin Treatment Silences Epithelial–Mesenchymal Transition Involved in Pulmonary Fibrosis in a Murine Model of Hypersensitivity Pneumonitis

**DOI:** 10.3390/ijms232113039

**Published:** 2022-10-27

**Authors:** Marta Kinga Lemieszek, Marcin Golec, Jacek Zwoliński, Jacek Dutkiewicz, Janusz Milanowski

**Affiliations:** 1 Department of Medical Biology, Institute of Rural Health, 20-090 Lublin, Poland; 2 Heidelberg Institute of Global Health (HIGH), Faculty of Medicine and University Hospital, Heidelberg University, 69117 Heidelberg, Germany; 3 Department of Biological Health Hazards and Parasitology, Institute of Rural Health, 20-090 Lublin, Poland; 4 Department of Pneumonology, Oncology and Allergology, Medical University of Lublin, 20-059 Lublin, Poland

**Keywords:** defense peptides, immune peptides, pulmonary fibrosis, hypersensitivity pneumonitis, extrinsic allergic alveolitis

## Abstract

Pulmonary fibrosis is becoming an increasingly common pathology worldwide. Unfortunately, this disorder is characterized by a bad prognosis: no treatment is known, and the survival rate is dramatically low. One of the most frequent reasons for pulmonary fibrosis is hypersensitivity pneumonitis (HP). As the main mechanism of pulmonary fibrosis is a pathology of the repair of wounded pulmonary epithelium with a pivotal role in epithelial–mesenchymal transition (EMT), we assumed that EMT silencing could prevent disease development. Because of several biological features including wound healing promotion, an ideal candidate for use in the treatment of pulmonary fibrosis seems to be cathelicidin. The aim of the studies was to understand the influence of cathelicidin on the EMT process occurring during lung fibrosis development in the course of HP. Cathelicidin’s impact on EMT was examined in a murine model of HP, wherein lung fibrosis was induced by chronic exposure to extract of *Pantoea agglomerans* (SE-PA) by real-time PCR and Western blotting. Studies revealed that mouse exposure to cathelicidin did not cause any side changes in the expression of investigated genes/proteins. Simultaneously, cathelicidin administered together or after SE-PA decreased the elevated level of myofibroblast markers (*Acta2*/α-smooth muscle actin, *Cdh2*/N-cadherin, *Fn1*/Fibronectin, *Vim*/vimentin) and increased the lowered level of epithelial markers (*Cdh1*/E-cadherin, *Ocln*/occludin). Cathelicidin provided with SE-PA or after cessation of SE-PA inhalations reduced the expression of EMT-associated factors (*Ctnnd1*/β-catenin, *Nfkb1*/NFκB, *Snail1*/Snail, *Tgfb1*/TGFβ1 *Zeb1*/ZEB1, *Zeb2*/ZEB2) elevated by *P. agglomerans*. Cathelicidin’s beneficial impact on the expression of genes/proteins involved in EMT was observed during and after the HP development; however, cathelicidin was not able to completely neutralize the negative changes. Nevertheless, significant EMT silencing in response to cathelicidin suggested the possibility of its use in the prevention/treatment of pulmonary fibrosis.

## 1. Introduction

Hypersensitivity pneumonitis (HP) or extrinsic allergic alveolitis (EAA) is a heterogenic group of interstitial lung diseases in which the chronic inhalation of a wide variety of organic dust provokes in susceptible subjects a hypersensitivity reaction with inflammation in the terminal bronchioles, the pulmonary interstitium, and the alveolar tree. This inflammation often organizes into granulomas and may progress to pulmonary fibrosis, which leads to the elimination of pathologically changed areas of lung tissue from the gas exchange, causing hypoxia and, in advanced cases, death [1,2,3]. HP can be provoked by a diverse range of antigens, including bacteria, fungi, mycobacteria, plant and animal proteins, chemicals, and metals [1]. Depending on the source and the type of antigens, several varieties of HP have been distinguished. One of the most common is farmer’s lung, induced by the inhalation of organic dust coming from agricultural products, mostly hay, straw, grain, and moldy plants, which are the source of a range of antigens, e.g., *Pantoea agglomerans*, *Saccharopolyspora rectivirgula*, *Streptomyces thermohygroscopicus*, *Streptomyces albus*, *Thermoactinomyces vulgaris*, *Thermoactinomyces viridis*, *Aspergillus fumigatus*, *Aspergillus niger*, *Absidia corymbifera*, and *Micropolyspora faeni*. Other very common types of HP are bird fancier’s lung (antigens: avian feathers, droppings, and serum); lung of mushroom growers (antigens: *Thermoactinomyces vulgaris*, *Micropolyspora faeni*); grain fever (antigens: *Pantoea agglomerans*, *Sitophilus granaries*); cheese disease (antigens: *Penicillium casei*, *Acarus siro*); and humidifier lung (antigens: *Alternaria alternata*, *Aureobasidium* spp., *Aspergillus* spp., *Bacillus* spp., *Cephalosporium* spp., *Fusarium* spp., *Trochoderma viridae*) [4,5]. Although the above-mentioned examples of different varieties of HP may suggest the presence of HP-associated environments, it has to be noted that extrinsic allergic alveolitis is caused by similar antigens in distinct environments, e.g., in differs ranges of agricultural environments in eastern Poland, one of the most important causes of HP is *Pantoea agglomerans* [5]. Nevertheless, because of the great variety and distribution of HP-induced antigens, millions of individuals are exposed to them as part of their occupational, home, or recreational environments. Thus, HP is estimated to be one of the most frequent reasons for pulmonary fibrosis with the known etiology; nevertheless, its global prevalence is relatively rare, especially when compared with idiopathic pulmonary fibrosis [6,7].

Emerging evidence suggests that pulmonary fibrosis is the pathology of respiratory repair following chronic lung epithelial injury [3,8,9,10,11]. In the case of hypersensitivity pneumonitis (HP), repeated injuries of the respiratory epithelium caused by chronic organic dust exposure leads to disorders of tissue repair. Successful wound repair requires close coordination of epithelial cell proliferation, migration, and differentiation with mesenchymal cell recruitment, proliferation, differentiation, and subsequent extracellular matrix remodeling. Deregulation of wound repair response leads to pathological scar formation and excessive deposition of extracellular matrix components, which rebuild and destroy normal tissue architecture [12,13,14]. Extracellular matrix deposition under physiological and pathological conditions is regulated primarily by myofibroblasts, which combine the features of fibroblasts and smooth muscle cells. These spindle-shaped cells produce a diverse range of cytokines, growth factors, and extracellular matrix components [12,13,15]. Several studies revealed that the most important source of myofibroblasts is an epithelial–mesenchymal transition (EMT). EMT describes the global process during which epithelial cells undergo local conversion, including loss of cell–cell adhesion and apical–basal polarity, and gain a mesenchymal phenotype including elongated shape, enhanced motility and invasiveness, and increased production of extracellular matrix [12,16,17,18]. Among three different types of EMT, a pivotal role in tissue regeneration and organ fibrosis is played by EMT type 2 [12,17]. EMT type 2 begins as a part of a normal repair-associated event that generates fibroblasts, myofibroblasts, and other related cells in order to reconstruct tissues following injury. EMT type 2 is linked to inflammation and, in the case of “physiological” repair, this process ceases once inflammation is attenuated. This is the signal that the wound is closed. In the case of pulmonary fibrosis, EMT type 2 and inflammation are ongoing, coupled in a vicious circle, until the fibrotic process reaches the point where it cannot be attenuated by calming down the inflammation [3,12,17]. The EMT initiation is triggered by cellular signaling mechanisms including Wnt/β-catenin and TGFβ pathways [12,17,18,19,20]. N-cadherin, α-SMA, vimentin, and fibronectin have been shown to be reliable biomarkers characterizing mesenchymal products generated by the EMT process occurring during fibrosis development. Additionally, E-cadherin and occludins were proven to be useful in the identification of epithelial cells undergoing an EMT associated with chronic inflammation [12,17]. Other studies demonstrated that EMT in lungs was characterized by downregulation of E-cadherin, occludins, cytokeratin, and aquaporins, while α-SMA, vimentin, collagens, N-cadherin, fibronectin, and desmin were upregulated [21,22,23,24]. Snai1, a direct transcriptional repressor for the E-cadherin gene, was demonstrated as a target for EMT-promoting signaling pathways [25]. Recently, additional transcription factors ZEB1 and ZEB2 were identified as E-cadherin repressors and mediators of the EMT [26]. Our earlier studies [27], conducted in mice strain C57BL/6J chronically exposed to the antigen of *Pantoea agglomerant* (well-documented etiological factor of HP) [5], have also shown downregulation of epithelial markers (*Cdh1*, *Cldn1*, *Jup*, *Ocln*) and upregulation of myofibroblasts markers (*Acta2*, *Cdh2*, *Fn1*, *Vim*) in lung tissue in response to bacterial antigen treatment. Furthermore, the mentioned alterations in gene expression typical for EMT correlated with an increase in fibrosis markers (hydroxyproline, collagens) as well as histological changes characteristic for lung fibrosis development [27]. Considering the role of EMT in pulmonary fibrosis [24,28,29,30], including the above-mentioned studies conducted by the authors that indicated EMT significance for the HP development in a murine model [27], it seemed reasonable to base HP therapy on inhibiting/counteracting EMT. Because of pleiotropic activities, an ideal candidate for the proposed therapeutic strategy seemed to be cathelicidin.

Cathelicidins belong to a large, conserved group of antimicrobial peptides and represent an important part of innate immunity [31]. These host defense peptides directly kill bacteria as well as some enveloped viruses, parasites, and fungi by perturbing their cell membranes [32,33]. Furthermore, they can also neutralize the biological activities of endotoxin [34,35]. Cathelicidins increase the natural abilities of immune cells to fight infection in several different ways, including attraction and recognition of pathogens, enhancement of phagocytosis, and stimulation of production and release of pro-inflammatory compounds [32,36,37,38,39,40]. Cathelicidins also accelerate epithelial cell proliferation, migration, and promotion of wound closure which, all together, play an important role in the maintenance of tissue homeostasis by supervising regenerative processes [41,42,43]. It needs to be highlighted that recent studies by the authors revealed the beneficial impact of cathelicidin on the development of lung fibrosis in the course of HP, which was associated with restoring the balance in quantity of immune cells (NK cells, macrophages, lymphocytes: Tc, Th, Treg, B), cytokine production (IFNγ, TNFα, TGFβ1, IL1β, IL4, IL5, IL10, IL12α, IL13), and synthesis of extracellular matrix components (hydroxyproline, collagens) [44]. Cathelicidin treatment also effectively protected lung tissue structure from pathological changes induced by antigen of *P. agglomerans* (HP trigger) [44]. Despite the fact that many advantageous biological activities of cathelicidin have been discovered, the presented study is the first to focus on understanding the influence of cathelicidin on the EMT process occurring during lung fibrosis development in the course of HP. Thus, the aim of the presented study was the identification of the molecular mechanism responsible for the antifibrotic properties of cathelicidin previously described by our research team [44].

## 2. Results

### 2.1. Cathelicidin Restored the Balance in the Expression of Genes Responsible for EMT

**Figure 1:**
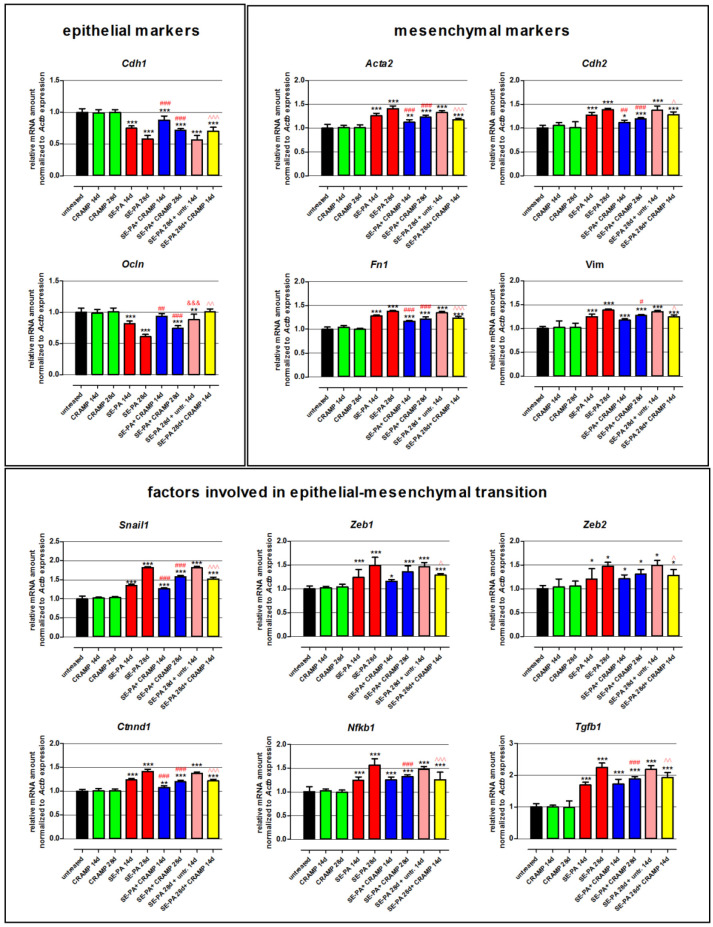
Alterations in the expression of genes involved in epithelial–mesenchymal transition (EMT) in response to cathelicidin (CRAMP) and/or saline extract of *Pantoea agglomerans* (SE-PA) treatment. Expression of gene coding epithelial markers, mesenchymal markers, and factors involved in epithelial–mesenchymal transition. Gene expression was investigated in homogenates of lungs collected from untreated mice (control) and animals exposed to investigated compounds for 14, 28, or 42 days using the real-time PCR method. Results are presented as the mean of relative mRNA amount ± SD. Each research group consisted of 8 mice: 6 treated and 2 untreated animals. Samples were collected from all animals and analyzed in 3 replications. Statistical significance from a one-way ANOVA test followed by Tukey’s post hoc test: compared to untreated mice at *p* < 0.05 (*), *p* < 0.01 (**), *p* < 0.001 (***); SE-PA + CRAMP 14 d/28 d vs. SE-PA 14 d/28 d (comparison within corresponding time points) at *p* < 0.05 (#), *p* < 0.01 (##), *p* < 0.001 (###); SE-PA 28 d + CRAMP 14 d vs. SE-PA 28 d + untreated 14 d at *p* < 0.05 (^), *p* < 0.01 (^^), *p* < 0.001 (^^^); SE-PA 28 d + untreated 14 d vs. SE-PA 28 d at *p* < 0.001 (&&&).

Changes in the expression of genes involved in epithelial–mesenchymal transition in lung tissue homogenates obtained from mice chronically exposed to cathelicidin and/or saline extract of *P. agglomerans* were examined by real-time PCR (Figure 1, Table 1). The study revealed that chronic exposure of mice to cathelicidin did not cause any changes in the expression of all investigated genes. On the contrary *P. agglomerans* treatment induced alterations in the gene expression characteristic for epithelial–mesenchymal transition: downregulation of epithelial markers (*Cdh1*, *Ocln*), as well as significant upregulation of mesenchymal markers (*Cdh2*, *Acta2*, *Fn1*, *Vim*). The decrease in epithelial markers, on average, was 21.6% after 14 days and 40.6% after 28 days of SE-PA exposure, while the increase in mesenchymal markers, on average, was 26.7% and 39.1% at the mentioned time points, respectively. The level of mRNA coding transcription factors responsible for EMT (*Snail1*, *Zeb1*, *Zeb2*) was also upregulated by SE-PA exposure; however, statistically significant changes were noted in the case of *Snail1* at both investigated time points (increase by 35.4% and 82.1%, respectively), and just after 28 days of inhalation with SE-PA in the case of *Zeb1* and *Zeb2* (average increase by 48.5%). Expression of representatives of signaling pathways leading to mesenchymal differentiation (*Ctnnd1*, *Nfkb1*, *Tgfb1*) in response to *P. agglomerans* reached the following levels: 1.236, 1.249, and 1.698 (2 weeks of inhalations) and 1.415, 1.565, and 2.255 (4 weeks of inhalations). The levels of almost all the investigated mRNA observed in mice 14 days after cessation of SE-PA chronic exposure were quite similar to the data obtained from mice only after 28 days of inhalation with *P. agglomerans*; the exception was *Ocln*, whose expression increased by 27.3% during 2 weeks without exposure. Cathelicidin administered together with SE-PA and after SE-PA treatment significantly increased the expression level of *Cdh1* and *Ocln* lowered by *P. agglomerans*. Expression of other investigated mRNAs recorded in the homogenates of lungs collected from mice treated with both CRAMP and SE-PA was lower compared with samples obtained from animals exposed to bacterial extract; nevertheless, differences observed in the case of *Zeb1* and *Zeb2* in both tested time points, as well as changes recorded in the case of *Vim*, *Nfkb1*, and *Tgfb1* after 14 days of exposure were not statistically significant. Statistically significant differences in the expression of *Cdh2*, *Acta2*, *Fn1*, *Snail*, and *Ctnnd1* recorded during comparison “SE-PA + CRAMP 14d.” vs. “SE-PA 14d.”, on average, were 13.1%, while in the expression of *Cdh2*, *Acta2*, *Fn1*, *Vim*, *Snail*, *Ctnnd1*, *Nfkb1*, and *Tgfb1* recorded during comparison of “SE-PA + CRAMP 28d.” vs. “SE-PA 28d.”, on average, were 21.1%. The cathelicidin administration after 28 days of mice exposure to SE-PA also decreased the elevated by *P. agglomerans* treatment level of all tested mesenchymal markers (on average by 11.7%), as well as factors involved in EMT (on average by 22.0%). It has to be noted that cathelicidin administered after cessation of SE-PA treatment better restored the balance in the mRNA levels of *Snail*, *Zeb1*, and *Zeb2*; on the contrary, alterations induced by *P. agglomerans* in the expression of other investigated genes better neutralized CRAMP together with bacterial extract. However, significant differences (at least 5%) were noted in the cases of *Cdh2*, *Ctnnd1*, *Fn1*, and *Tgfb1*.

### 2.2. Cathelicidin Eliminated Negative Changes in the Expression of Proteins Associated with EMT

**Figure 2:**
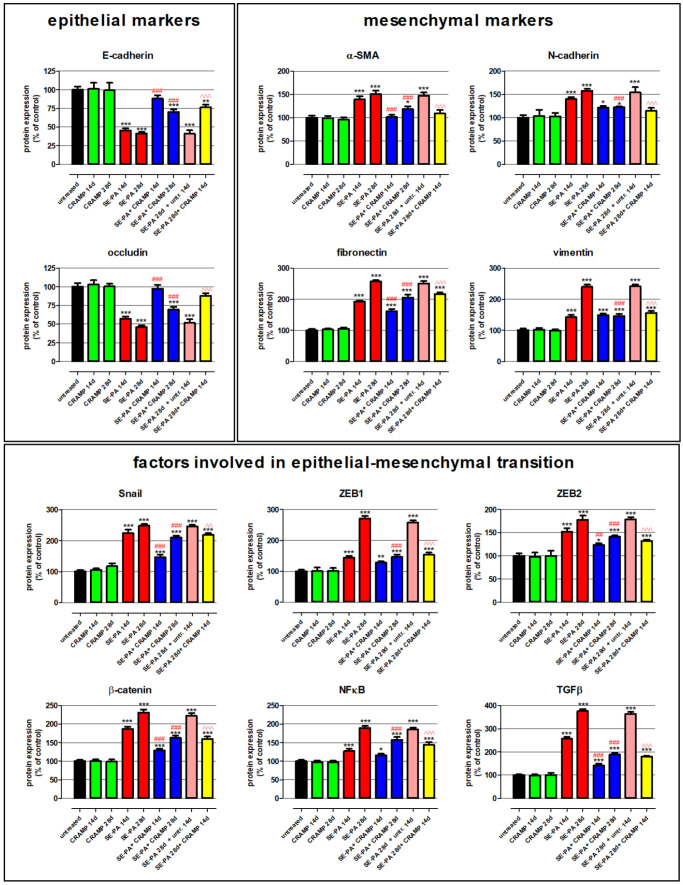

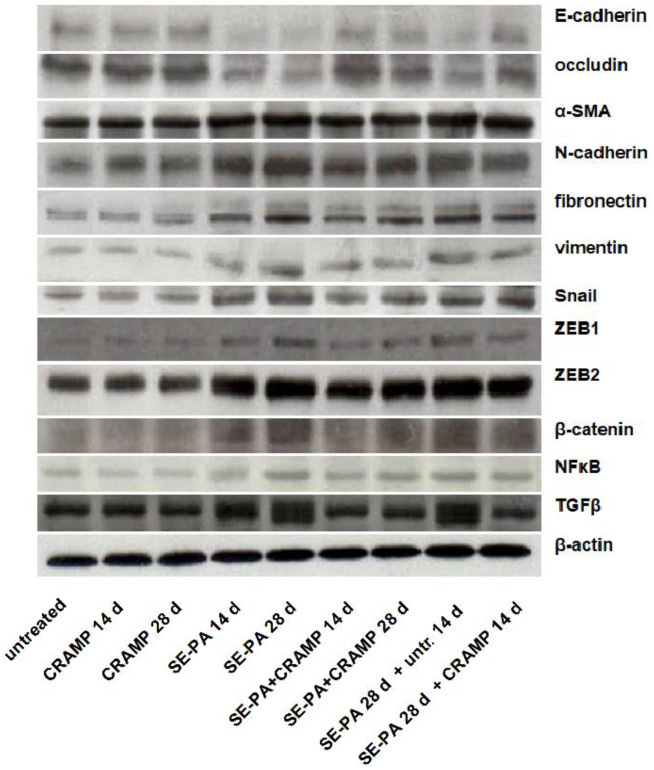
Alterations in the expression of proteins involved in epithelial–mesenchymal transition (EMT) in response to cathelicidin (CRAMP) and/or saline extract of *Pantoea agglomerans* (SE-PA) treatment. Protein expression was investigated in homogenates of lungs collected from untreated mice (control) and animals exposed to investigated compounds for 14, 28, or 42 days using the Western blotting method. Examination of ß-actin expression level was used as the internal control. Representative Western blots with densitometric analyses of epithelial markers, mesenchymal markers, and factors involved in epithelial–mesenchymal transition. Results of the densitometric analysis are presented as the mean of protein expression ± SD. Each research group consisted of 8 mice: 6 treated and 2 untreated animals. Samples collected from animals belonging to the common research group were mixed in equal volumes and then analyzed in 3 replications. Statistical significance from one-way ANOVA test followed by Tukey’s post hoc test: compared to untreated mice at *p* < 0.05 (*), *p* < 0.01 (**), *p* < 0.001 (***); SE-PA + CRAMP 14 d/28 d vs. SE-PA 14 d/28 d (comparison within corresponding time points) at *p* < 0.01 (##), *p* < 0.001 (###); SE-PA 28 d + CRAMP 14 d vs. SE-PA 28 d + untreated 14 d at *p* < 0.01 (^^), *p* < 0.001 (^^^).

Changes in the expression of proteins involved in epithelial–mesenchymal transition were investigated by Western blotting in homogenates of lung tissue collected from mice treated with cathelicidin and/or saline extract of *Pantoea agglomerans* (Figure 2, Table 2). Western blots revealed that cathelicidin used alone did not impact the expression of proteins associated with EMT: E-cadherin, N-cadherin, β-catenin, fibronectin, NFκB, vimentin, occludin, Snail, α-smooth muscle actin (α-SMA), TGFβ, ZEB1, ZEB2. On the contrary, chronic exposure of mice to saline extract of *P. agglomerans* significantly decreased the expression of epithelial markers (E-cadherin, occludin) and distinctly increased the level of mesenchymal markers (N-cadherin, fibronectin, vimentin, α-SMA). Expression of epithelial markers were lowered, on average, 48.8% and 56.4% after 14 and 28 days of SE-PA exposure, respectively. Simultaneously, the expression of mesenchymal markers grew, in response to 14 and 28 days of mice treatment with *P. agglomerans*, on average by 53.7% and 101.4%, respectively. Furthermore, a distinct increase in transcription factors responsible for EMT (Snail, ZEB1, ZEB2) and key members of signaling pathways involved in EMT (β-catenin, NFκB, TGFβ) was also observed in SE-PA-treated mice. The most significant changes were recorded in the case of TGFβ, in which expression on the 14th and 28th days of the experiment reached 257.3% and 376.0% of the control, respectively. Equally important changes were observed in the case of Snail and β-catenin, whose expressions increased to 224.2% and 247.6% of the control at the above-mentioned time points, respectively. It has to be noted that the expression of all investigated proteins recorded at 28 days of SE-PA treatment and 14 days after cessation of SE-PA chronic exposure was maintained at a similar level. Cathelicidin administered together with SE-PA and after cessation of SE-PA treatment significantly increased the expression of epithelial markers lowered by inhalations with *P. agglomerans*, and the most significant improvement was observed in the case of occludin, the expression of which after 14 days of CRAPM + SE-PA exposure approached the level of the control. The expression of almost all mesenchymal markers (except vimentin in time point of 14th days) elevated by the bacterial extract was significantly lower in the lung samples collected from mice treated simultaneously with SE-PA and CRAMP as well as animals treated with defense peptide for the next 14 days after cessation of antigen administration. The most spectacular restoration of the level of mesenchymal markers altered by SE-PA was observed in the case of α-SMA, the expression of which after 14 days of cathelicidin administration together with SE-PA dropped to the level registered in untreated mice; the differences between the compared research groups (SE-PA + CRAMP 14d vs. SE-PA 14d) was 38.5%. The beneficial effect of cathelicidin given together with or after *P. agglomerans* treatment was also observed in the expression of factors involved of EMT, the level of which significantly decreased, compared to the data recorded in lung samples collected from mice treated with SE-PA. Among the investigated factors, the most significant improvement was observed in the expression of TGFβ, the level of which decreased by 115.8% (SE-PA + CRAMP 14d vs. SE-PA 14d), 187.4% (SE-PA + CRAMP 28d vs. SE-PA 28d), and 185.0% (SE-PA 28d + CRAMP 14d vs. SE-PA 28d + untreated 14d). The second factor with the highest amplitude of changes was ZEB1, the level of which decreased by 124.3% (SE-PA + CRAMP 28d vs. SE-PA 28d) and 103.7% (SE-PA 28d + CRAMP 14d vs. SE-PA 28d + untreated 14d); however, the difference of 16.7% in the expression of ZEB1 observed between “SE-PA + CRAMP 14d” and “SE-PA 14d” was not statistically significant. The beneficial impact of cathelicidin administered together or after *P. agglomerans* exposure on the expression of investigated proteins altered by SE-PA was quite similar in most of the performed experiments. Nevertheless, in the case of Snail and β-catenin, cathelicidin worked much better if given together with SE-PA; on the contrary, the defense peptide administered after cessation of SE-PA exposure better restored the balance in the expression of ZEB2, NFκB, and N-cadherin.

## 3. Discussion

Despite the well-described EMT role in pulmonary fibrosis [24,27,28,29,30,45], as well as several pieces of data indicating cathelicidin involvement in the EMT process [41,42,43,46], there is no evidence regarding the cathelicidin influence of EMT in the course of HP development. The current study fills this gap of knowledge, describing the cathelicidin impact on the expression of genes and proteins involved in EMT under developing lung fibrosis in the course of HP. The study was conducted in created and validated by our research group the murine model of HP, wherein pulmonary fibrosis was induced by an extract of *Pantoea agglomerans* administered daily for 28 days to fibrosis mice strain C57BL/6J [47,48,49,50,51]. Pathological changes observed in mice in response to chronic exposure to the antigen of *P. agglomerans* were similar to the clinical picture of HP, which was confirmed on the genome and proteome level as well as in the microscopic image of lung tissue and in the characteristics of the immune response. Furthermore, earlier studies by our team also revealed that depending on the time of exposure, successive stages of disease development can be obtained: acute with a strong inflammatory response (7–14 days of exposure) and chronic with significant signs of fibrosis (28 days of exposure) [47,48,49,50,51]. It should be emphasized that according to our best knowledge, the mentioned model is the only research model of HP that, under laboratory conditions, reproduces the environmental exposure to organic dust causing lung fibrosis. For this reason, the use of this model in the presented study increases the chances of effective translation of the obtained results into clinical practice, thus accelerating the introduction of a new HP therapeutic strategy. An additional reason for selecting the mentioned model for the current study was the previously mentioned data [27], which demonstrated the relationship between pulmonary fibrosis induced by mice chronic exposure to *P. agglomerans* with the antigen ability to provoke epithelial–mesenchymal transition type 2, which is the focus of this study. First of all, the study revealed that the chronic exposure of mice to cathelicidin did not cause any changes in the expression of all investigated molecules, neither the genes nor their proteins products: *Acta2*/α-smooth muscle actin, *Cdh1*/E-cadherin, *Cdh2*/N-cadherin, *Ctnnd1*/β-catenin, *Fn1*/fibronectin, *Nfkb1*/NFκB, *Vim*/vimentin, *Ocln*/occludin, *Snail1*/Snail, *Tgfb1*/TGFβ, *Zeb1*/ZEB1, *Zeb2*/ZEB2. On the contrary, SE-PA treatment induced alterations characteristic for EMT: downregulation of epithelial markers (*Cdh1*/E-cadherin, *Ocln*/occludin) and significant upregulation of mesenchymal markers (*Cdh2*/N-cadherin, *Fn1*/fibronectin, *Vim*/vimentin, *Acta2*/α-smooth muscle actin). Chronic exposure of mice to *P. agglomerans* also increased the level of transcription factors responsible for the EMT process (*Snail1*/Snail, *Zeb1*/ZEB1, *Zeb2*/ZEB2) and selected members of signaling pathways, leading to mesenchymal differentiation (*Ctnnd1*/β-catenin, *Nfkb1*/NFκB, *Tgfb1*/TGFβ). The most significant changes were noted in the expressions of *Tgfb1*/TGFβ and *Snail1*/Snail. The observed changes highlight the key roles of these two molecules in the execution of the EMT program in response to SE-PA exposure. TGFβ has been shown to play a pivotal role in pulmonary fibrosis, not only through induction of EMT in alveolar epithelial cells and its ability to attract and stimulate proliferation of fibroblasts and myofibroblasts but also as potent inducers of ECM production, including collagen and other matrix proteins [28,29,52,53]. Since *Snail* is an immediate-early response gene for TGFβ, the observed significant increase in its expression seems to be logical. Indeed, most EMT studies revealed Snail induction in response to TGFβ and furthermore demonstrated a correlation between the elevated level of this transcription factor with the repression of genes coding epithelial markers, as well as concomitant activation of mesenchymal gene expression [54,55,56]. While the amplitudes of changes in the level of expression of other investigated genes in response to SE-PA were similar, analysis of protein expression indicated two additional molecules (ZEB1 and β-catenin) that, in addition to the TGFβ and Snail, were proven to be very sensitive to the action of the tested bacterial extract. As in the case of the Snails, the expression of transcription factor ZEB1 is activated by TGFβ and directly represses the expression of epithelial marker genes, increases expression of the mesenchymal markers, and additionally promotes cell migration [29,54,57,58,59]. β-catenin is physiologically part of a major cell-surface adhesion complex, but if released from there becomes an important part of Wnt signaling, the involvement of which in EMT has been proven in many studies [29,30,57]. Released β-catenin interacts with transcription factors LEF1 or TCF and as a complex translocates into the nucleus and regulates the transcription of several genes associated with EMT, e.g., decreasing the expression of E-cadherin gene, increasing the expression of genes coding vimentin and fibronectin, and stimulating the production of matrix metalloproteinases [30,57,60].

The conducted studies revealed that cathelicidin administered with SE-PA or after 28 days of *P. agglomerans* exposure restored the balance in the expression of genes and proteins disturbed by mice exposure to the mentioned extract. CRAMP increased the lowered by SE-PA level of *Cdh1*/E-cadherin and *Ocln*/occludin. Cathelicidin particularly effectively neutralized the negative effect of *P. agglomerans* on the expression of *Ocln*/occludin—14 days of CRAMP treatment together with SE-PA or after cessation of SE-PA inhalations increased the expression of the investigated molecule to the level observed in untreated mice. Additionally, CRAMP administered with bacterial extract or after SE-PA exposure decreased the elevated by SE-PA level of *Cdh2*/N-cadherin, *Acta2*/α-SMA, *Fn1*/fibronectin, and *Vim*/vimentin. Nevertheless, complete neutralization of the alteration induced by SE-PA was observed in the case of α-SMA after 14 days of mice exposure to both CRAMP and SE-PA. The similar beneficial impact of the tested peptide on α-SMA expression was noted in mice after 14 days of CRAMP treatment preceded by 28 days of SE-PA inhalations. Alterations in the expression of factors associated with EMT observed in mice chronically exposed to SE-PA were also leveled by cathelicidin treatment. Nevertheless, the effectiveness of cathelicidin treatment depends on the way of cathelicidin administration. The investigation of gene expression revealed three different patterns of CRAMP response: (1) *Snail1* and *Ctnnd1*: significant differences in the expression after 14 and 28 days of animals exposure to SE-PA used alone and together with CRAMP; (2) *Nfkb1* and *Tgfb*: significant differences in the expression after 28 days of animals exposure to SE-PA used alone and together with CRAMP; (3) differences in the expression of *Zeb1* and *Zeb2* after 14 and 28 days of animals exposure to SE-PA used alone and together with CRAMP were not significant. Nevertheless, alteration in the expression of all investigated factors was restored when CRAMP was provided after cessation of SE-PA inhalations. Results obtained from Western blotting revealed that mice inhalation with CRAMP after 28 days of SE-PA exposure as well as CRAMP administration for 4 weeks together with SE-PA significantly lowered the pathological level of all investigated factors. Furthermore, the expression of Snail, ZEB2, β-catenin, and TGFβ was also improved in mice exposed to both CRAMP and *P. agglomerans* antigene for 14 days.

It should be emphasized that changes recorded on genome and proteome levels correspond with alterations in lung tissue morphology previously described by our team [44] (see the Supplementary Materials Figure S1). Similar to the presented data, histological examination of murine lungs stained with hematoxylin and eosin (H&E) or Masson trichrome (TRI) revealed the lack of any changes in tissue morphology after 14 and 28 days of animal exposure to cathelicidin. On the contrary, mice inhalations with SE-PA induced changes typical for HP; in particular, significant inflammatory response with interstitial infiltrations of lymphocyte and macrophage (mean score for inflammation in time points 14 and 28: 1.6 and 2.0) as well as signs of fibrosis (mean score for fibrosis in time points 14 and 28: 0.8 and 1.6) associated with abnormal collagen deposition, leading to thickening of alveolar walls, which intensified during the time of exposure. The mentioned alterations perfectly corresponded with the herein presented increase in expression of mesenchymal markers and transcription factors involved in EMT. Moreover, the histological examination also did not show statistically significant changes between lungs collected 14 days after cessation of mice chronic exposure to SE-PA to lungs obtained directly after 28 days of inhalation with *P. agglomerans* (SE-PA 28d + untreated 14d vs. SE-PA 28d). Furthermore, the evaluation of lung sections also revealed the beneficial impact of cathelicidin treatment on fibrosis development. Comparison data obtained from the following research groups: SE-PA + CRAMP 28d vs. SE-PA 28d as well as SE-PA 28d + CRAMP 14d vs. SE-PA 28d + untreated 14d, demonstrated a significant decrease in fibrosis scores by 36.8% and 38.9%, respectively. The above-mentioned antifibrotic properties of cathelicidin correspond with data obtained in presented studies, that revealed the beneficial impact of CRAMP treatment in the inhibition of the expression of both mesenchymal markers and factors involved in EMT, which have been pathologically elevated by *P. agglomerans*. It needs to be highlighted that the concordance of changes observed at the level of genes and proteins as well as in the histological evaluation indicated that the previously described beneficial properties of cathelicidin should be associated with its ability to modulate the course of EMT [44].

The presented results indicate CRAMP as an effective inhibitor of EMT associated with pulmonary fibrosis in HP, which is the first such evidence according to the best of our knowledge. Nevertheless, the ability of cathelicidin to inhibit pathological EMT was reported previously by Cheng et al., who showed that LL-37 inhibited TGFβ-induced gene expression of *ACTA2* and *VIM* as well as protein expression of E-cadherin, Twist1, and Slug in human colon cancer HT-29 cells. Furthermore, Cheng et al. demonstrated in vivo that administration of cathelicidin expression viral vector or synthetic mCRAMP inhibited vimentin and E-cadherin expression and collagen deposition, leading to suppression of EMT and consequently to colon cancer development [46]. Additionally, Zheng et al. revealed the inhibition of cardiac fibrosis in diabetic mice heart treated with CRAMP, which was a consequence of an increase in endothelial markers (CD31, cadherin), a decrease in fibroblast markers (collagen I, collagen III, vimentin), and a reduction of transcription factors (Snial1, Snial2, Twist1, Twist2). Zheng et al. connected the anti-fibrotic CRAMP abilities with the silencing of signal transduction in the TGFβ/Smad pathway [61]. Despite the fact that some alterations in the expression of genes and proteins in response to CRAMP observed by Cheng et al. and Zheng et al. correspond with the currently presented results, it has to be stressed that there are differences. Cheng et al. investigated the impact of cathelicidin on EMT type 3 [46], while Zheng et al. discovered the beneficial impact of CRAMP on endothelial–mesenchymal transition [61]. Consequently, the presented study is the first report showing the influence of cathelicidin on EMT type 2. Furthermore, obtained data indicated that maintaining the physiological level of cathelicidin in the respiratory tract plays an important role in the supervision of regenerative processes and consequently prevention of pathological wound healing leading to fibrosis.

## 4. Materials and Methods

### 4.1. Reagents

Unless otherwise indicated, the chemicals used in the study were purchased from Sigma-Aldrich Co. (St. Louis, MO, USA) LLC. Murine cathelicidin (ISRLAGLLRKGGEKIGEKLKKI GQKIKNFFQKLVPQPE) was purchased from Novozym Polska s.c. Poznań Science and Technology Park, Poznań, Poland. The preparation of Pantoea agglomerans extract, as well as its main composition, has been described previously [27].

### 4.2. Animal Inhalations

Three-month-old female C57BL/6J mice were purchased from Mossakowski Medical Research Centre of the Polish Academy of Sciences in Warsaw, Poland. The conditions in which the animals were kept, as well as the procedure for their preparation for the study, has been described previously [44]. Mice were exposed to a finely dispersed aerosol of the saline extract of *P. agglomerans* (SE-PA; 10 mg/mL; 5 mL/single inhalation), cathelicidin (CRAMP; 1.44 µg/mL; 5 mL/single inhalation), or phosphate-buffered saline (PBS; 5 mL/single inhalation), administered separately or in combination (Table 3). The mice were treated to each investigated factor for one hour daily for 14, 28, or 42 days. Inhalations were carried out using the Buxco Inhalation Tower (Data Sciences International, St. Paul, MN, USA) under the following conditions: airflow 1.5 L/min; pressure 0.5 cm H_2_O; room temperature; nebulization rate 84 μL/min. Before and after the indicated time of treatment, the animals were sacrificed by cervical dislocation with spinal cord rupture, after which lung samples were collected, frozen in liquid nitrogen, and stored at −80 °C until evaluation.

### 4.3. Evaluation of Gene Expression

Gene expression evaluation has been described previously [62]; nevertheless, what should be mentioned are the TaqMan Gene Expression Assays (sets of probes and primers) used in the research: Mm00725412_s1 for *Acta2*; Mm02619580_g1 for *Actb*; Mm01247357_m1 for *Cdh1*; Mm01162497_m1 for *Cdh2*; Mm01334599_m1 for *Ctnnd1*; Mm01256744_m1 for *Fn1*; Mm00476361_m1 for *Nfkb1*; Mm00500912_m1 for *Ocln*; Mm01178820_m1 for *Tgfb1*; Mm00441533_g1 for *Snail1*; Mm01333430_m1 for *Vim*; Mm00495564_m1 for *Zeb1*; Mm00497196_m1 for *Zeb2*. Relative expression was calculated using the efficiency method (relative advanced quantification) and normalized to the expression of *Actb*.

### 4.4. Evaluation of Proteins Expression

Examination of protein expression has been described previously [62]. The investigation has been conducted using primary antibodies directed against β-actin, β-catenin, E-cadherin, N-cadherin, Snail, vimentin (Cell Signaling Technology, Danvers, MA, USA), α-smooth muscle actin, fibronectin, occludin, NFκB, TGFβ, ZEB1, and ZEB2 (ThermoFisher Scientific, Waltham, MA, USA). The amount of protein was densitometrically determined using ImageJ software.

### 4.5. Statistical Analysis

The data were presented as the mean value and standard deviation (SD). Statistical analysis was performed using linear regression analysis, as well as the one way-ANOVA with Tukey’s post hoc test, and column statistics were used for comparisons. Significance was accepted at *p* < 0.05.

## 5. Conclusions

In summary, the beneficial impact of cathelicidin treatment on the expression of genes/proteins involved in EMT was observed both during and after the development of hypersensitivity pneumonitis. Despite the fact that cathelicidin was not able to completely neutralize the negative changes induced by *P. agglomerans*, EMT silencing in response to CRAMP was significant. Furthermore, cathelicidin used alone did not cause any side-changes in the expression of the investigated genes and proteins. It needs to be highlighted that the presented study is the first report showing the influence of cathelicidin on EMT type 2. Due to the importance of EMT for pulmonary fibrosis, the presented results suggest the possibility of using cathelicidin in the prevention and treatment of this pathological process. Unfortunately, exogenous cathelicidin was not able to eliminate the negative changes completely, perhaps because of the fact that CRAMP inhalations did not restore the physiological level of cathelicidin disturbed by the disease development. Nevertheless, in the face of the absence of a safe and successful strategy for the prevention and treatment of pulmonary fibrosis in the course of HP, even a slight beneficial effect of cathelicidin, especially in terms of the lack of side effects, deserves attention and presentation in order to create the basis for the development an effective therapeutic strategy in the future.

## Figures and Tables

**Table 1:** Expression of genes involved in EMT in response to cathelicidin (CRAMP) and/or saline extract of *Pantoea agglomerans* (SE-PA) treatment. Results are presented as the mean of relative mRNA amount ± SD.

	Untreated	CRAMP 14 d	CRAMP 28 d	SE-PA 14 d	SE-PA 28 d	SE-PA + CRAMP 14 d	SE-PA + CRAMP 28 d	SE-PA 28 d + Untreated14 d	SE-PA 28 d + CRAMP14 d
*Cdh1*	1.000 ± 0.058	0.988 ± 0.051	0.996 ± 0.042	0.748 ± 0.036	0.579 ± 0.059	0.873 ± 0.069	0.715 ± 0.024	0.566 ± 0.066	0.701 ± 0.061
*Ocln*	1.003 ± 0.064	0.986 ± 0.057	1.008 ± 0.062	0.820 ± 0.041	0.610 ± 0.035	0.936 ± 0.040	0.744 ± 0.042	0.883 ± 0.087	1.006 ± 0.051
*Acta2*	1.003 ± 0.075	1.010 ± 0.051	1.006 ± 0.065	1.261 ± 0.055	1.408 ± 0.058	1.121 ± 0.059	1.231 ± 0.046	1.329 ± 0.039	1.175 ± 0.032
*Cdh2*	1.003 ± 0.056	1.053 ± 0.065	1.005 ± 0.013	1.270 ± 0.065	1.389 ± 0.030	1.119 ± 0.051	1.200 ± 0.023	1.375 ± 0.092	1.280 ± 0.062
*Fn1*	1.001 ± 0.046	1.043 ± 0.034	0.995 ± 0.027	1.285 ± 0.014	1.376 ± 0.018	1.168 ± 0.018	1.216 ± 0.046	1.346 ± 0.029	1.233 ± 0.038
*Vim*	1.001 ± 0.043	1.020 ± 0.014	1.025 ± 0.094	1.253 ± 0.054	1.390 ± 0.029	1.181 ± 0.027	1.275 ± 0.018	1.355 ± 0.030	1.248 ± 0.037
*Snail1*	1.003 ± 0.069	1.023 ± 0.024	1.033 ± 0.028	1.354 ± 0.030	1.821 ± 0.027	1.264 ± 0.023	1.580 ± 0.035	1.813 ± 0.041	1.525 ± 0.047
*Zeb1*	1.003 ± 0.058	1.021 ± 0.034	1.046 ± 0.058	1.241 ± 0.165	1.490 ± 0.176	1.158 ± 0.044	1.355 ± 0.131	1.466 ± 0.088	1.288 ± 0.034
*Zeb2*	1.001 ± 0.073	1.043 ± 0.163	1.058 ± 0.106	1.210 ± 0.212	1.479 ± 0.081	1.214 ± 0.080	1.310 ± 0.098	1.495 ± 0.100	1.283 ± 0.125
*Ctnnd1*	1.003 ± 0.036	1.005 ± 0.052	1.004 ± 0.042	1.236 ± 0.030	1.415 ± 0.046	1.079 ± 0.031	1.201 ± 0.029	1.376 ± 0.029	1.218 ± 0.034
*Nfkb1*	1.005 ± 0.106	1.024 ± 0.037	0.996 ± 0.049	1.249 ± 0.066	1.565 ± 0.014	1.256 ± 0.056	1.324 ± 0.040	1.483 ± 0.055	1.258 ± 0.016
*Tgfb1*	1.005 ± 0.101	1.008 ± 0.053	0.983 ± 0.211	1.698 ± 0.090	2.255 ± 0.132	1.725 ± 0.153	1.886 ± 0.075	2.188 ± 0.112	1.928 ± 0.153

**Table 2:** Expression of proteins involved in EMT in response to cathelicidin (CRAMP) and/or saline extract of *Pantoea agglomerans* (SE-PA) treatment. Results are presented as the mean of protein expression ± SD.

	Untreated	CRAMP 14 d	CRAMP 28 d	SE-PA 14 d	SE-PA 28 d	SE-PA + CRAMP 14 d	SE-PA + CRAMP 28 d	SE-PA 28 d + Untreated14 d	SE-PA 28 d + CRAMP14 d
E-cadherin	100.0 ± 4.5	101.1 ± 8.4	99.4 ± 10.1	45.4 ± 2.7	41.2 ± 2.1	88.3 ± 4.4	70.2 ± 3.5	40.9 ± 4.8	76.3 ± 3.8
Occludin	100.0 ± 5.2	102.8 ± 6.0	100.4 ± 3.7	56.9 ± 3.1	46.0 ± 2.5	97.2 ± 5.4	69.4 ± 3.8	51.5 ± 5.2	87.9 ± 3.5
α-SMA	100.0 ± 5.0	98.9 ± 4.9	96.1 ± 4.8	139.5 ± 7.0	150.8 ± 7.5	101.3 ± 5.1	118.4 ± 6.3	147.3 ± 7.4	109.3 ± 7.9
N-cadherin	100.0 ± 5.6	103.1 ± 14.0	102.7 ± 7.7	140.2 ± 4.1	157.6 ± 4.2	121.6 ± 3.6	122.5 ± 2.3	154.5 ± 11.1	114.9 ± 6.3
Fibronectin	100.0 ± 4.5	102.8 ± 2.8	104.8 ± 4.6	192.8 ± 2.8	257.2 ± 4.2	161.4 ± 7.3	205.6 ± 9.3	251.0 ± 7.8	216.9 ± 4.8
Vimentin	100.0 ± 5.0	102.2 ± 5.1	98.1 ± 4.9	142.4 ± 7.1	240.0 ± 7.2	148.4 ± 5.2	145.6 ± 7.3	241.6 ± 5.9	155.9 ± 6.5
Snail	100.0 ± 5.0	105.0 ± 5.3	117.7 ± 9.2	224.2 ± 11.2	247.6 ± 5.8	146.6 ± 7.3	209.9 ± 6.2	246.1 ± 4.2	217.9 ± 5.7
ZEB1	100.0 ± 5.1	100.9 ± 11.4	101.5 ± 9.1	144.3 ± 5.7	270.8 ± 8.5	127.6 ± 4.6	146.5 ± 7.4	257.1 ± 8.1	153.3 ± 7.8
ZEB2	100.0 ± 5.0	98.2 ± 9.1	99.9 ± 11.1	152.0 ± 7.6	177.6 ± 9.0	123.6 ± 3.6	141.0 ± 3.0	178.6 ± 5.1	132.3 ± 2.8
β-catenin	100.0 ± 3.6	100.5 ± 4.8	98.9 ± 5.9	186.8 ± 6.7	231.4 ± 8.3	129.6 ± 4.6	163.2 ± 5.8	222.0 ± 7.9	160.1 ± 6.3
NFκB	100.0 ± 3.5	97.3 ± 4.9	97.4 ± 4.9	127.2 ± 6.4	189.6 ± 5.6	117.5 ± 5.9	157.7 ± 7.9	185.3 ± 5.8	144.2 ± 7.2
TGFβ	100.0 ± 4.0	97.9 ± 5.3	100.3 ± 9.7	257.3 ± 7.9	376.0 ± 8.6	141.5 ± 8.8	188.6 ± 7.6	364.7 ± 7.6	179.7 ± 3.1

**Table 3:** The research group description.

Name of Research Group	PBS (Time of Exposure)	SE-PA (Time of Exposure)	CRAMP (Time of Exposure)	Factors Administration Sequence
untreated (*n* = 16)	-	-	-	-
CRAMP 14 d (*n* = 6)	1 h a day for 14 days	-	1 h a day for 14 days	one by one on the same day
CRAMP 28 d (*n* = 6)	1 h a day for 28 days	-	1 h a day for 28 days	one by one on the same day
SE-PA 14 d (*n* = 6)	1 h a day for 14 days	1 h a day for 14 days	-	one by one on the same day
SE-PA 28 d (*n* = 6)	1 h a day for 28 days	1 h a day for 28 days	-	one by one on the same day
SE-PA + CRAMP 14 d (*n* = 6)	-	1 h a day for 14 days	1 h a day for 14 days	one by one on the same day
SE-PA + CRAMP 28 d (*n* = 6)	-	1 h a day for 28 days	1 h a day for 28 days	one by one on the same day
SE-PA 28 d + untreated 14 d (*n* = 6)	1 h a day for 28 days	1 h a day for 28 days	-	one by one on the same day
	-	-	-	After 28 days of treatment, mice stayed an additional 14 days in the experiment without exposure
SE-PA 28 d + CRAMP 14 d (*n* = 6)	1 h a day for 28 days	1 h a day for 28 days	-	one by one on the same day
			1 h a day for 14 days	after 28 days of mice exposure to (PBS + SE-PA), CRAMP was applied for an additional 14 days

PBS—phosphate-buffered saline; SE-PA—saline extract of Pantoea agglomerans; CRAMP—cathelicidin; *n*—number of animals in research group; d—days.

## Data Availability

All data analyzed during this study are included in this article. Further inquiries can be directed towards the corresponding author.

## Supplementary Materials

The supporting information can be downloaded at https://www.mdpi.com/article/10.3390/ijms232113039/s1.
