# Supplementary material for: Cathelicidin Treatment Silences Epithelial–Mesenchymal Transition Involved in Pulmonary Fibrosis in a Murine Model of Hypersensitivity Pneumonitis
Source: Int J Mol Sci. 2022 Oct 27;23(21):13039. doi: 10.3390/ijms232113039 (PMC9659202; doi:10.3390/ijms232113039)
Supplement: Supplementary file 1 [file ijms-23-13039-s001.zip › ijms-1980063-supplementary.pdf]

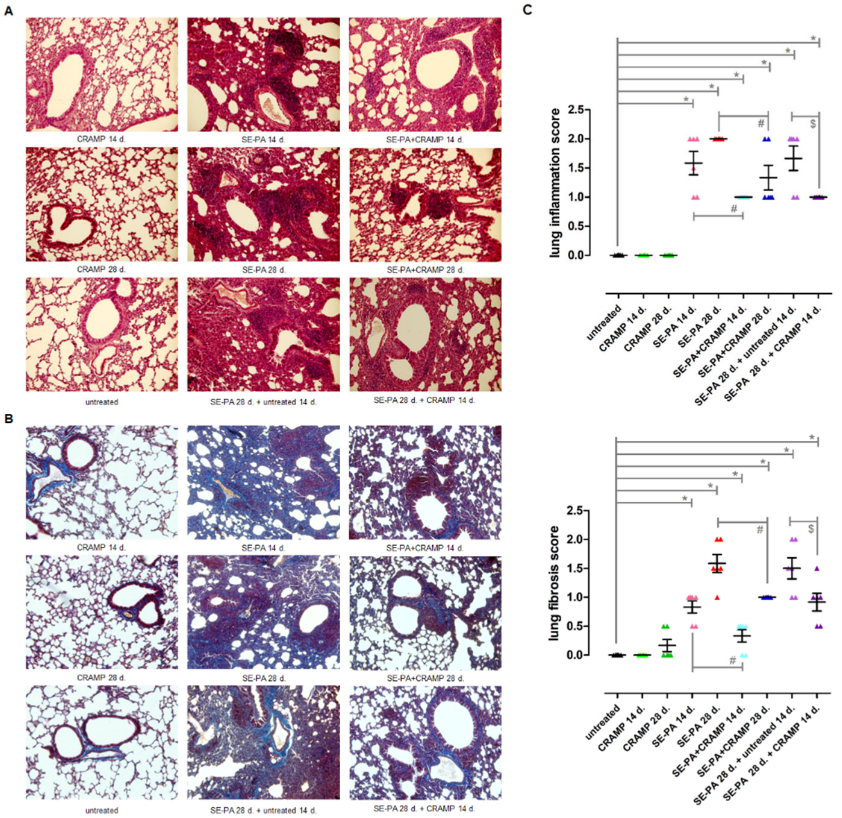

Figure S1. Changes in the morphology of lung tissue of mice exposed to cathelicidin (CRAMP) and/or saline extract of *Pantoea agglomerans* (SE-PA). Lungs collected from untreated mice and animals exposed to the investigated compounds for 14, 28, or 42 days were stained with hematoxylin and eosin (H&E), as well as Masson trichrome dyes (TRI), and examined under light microscopy at a magnification of 200×. Each research group contained 8 mice: 6 treated and 2 untreated animals. Samples were collected from all animals and analyzed (control mice N = 16; treated mice per research group N = 6). (**A**) Representative photographs of mouse lung sections stained with H&E. (**B**) Representative photographs of mouse lung sections stained with TRI. (**C**) Quantification of inflammation and fibrosis in collected mouse lung tissue after H&E and TRI staining. The histological scores were graded with 4-point scales: 0 = regular tissue, 1 = mild changes, 2 = moderate changes, 3 = significant changes. The data for histologic scores for 2 independent investigations are given as mean ± SEM of the investigated items. * $p < 0.05$ vs. untreated; # $p < 0.05$ SE-PA+CRAMP 14 d/28 d vs. SE-PA 14 d/28 d (comparison within corresponding time points); $ $p < 0.05$ SE-PA 28 d + CRAMP 14 d vs. SE-PA 28 d + untreated 14 d; *on*e-way ANOVA test; Tukey's post hoc test. The figure was originally published in [44].
